# Supplementary material for: Hyaluronan and Derivatives: An In Vitro Multilevel Assessment of Their Potential in Viscosupplementation
Source: Polymers (Basel). 2021 Sep 22;13(19):3208. doi: 10.3390/polym13193208 (PMC8512809; doi:10.3390/polym13193208)
Supplement: Supplementary file 1 [file polymers-13-03208-s001.zip › polymers-1340051-supplementary.pdf]

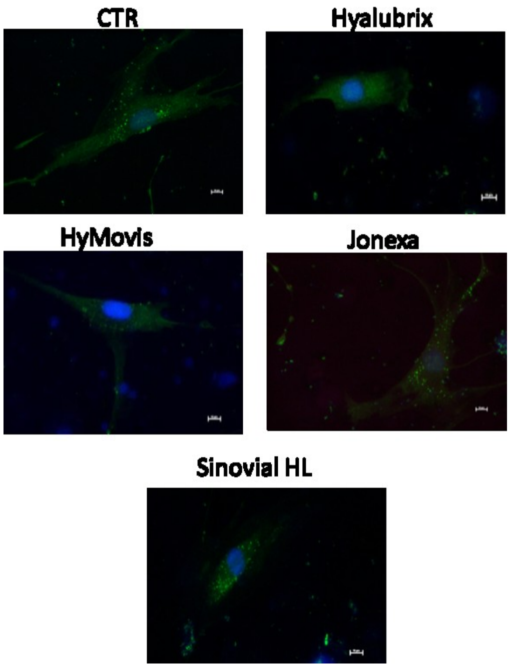

Figure S1 a. Immunofluorescence staining of COMP-2 in treated and untreated primary human chondrocytes. In the blue nuclei, a fitc-green antibody was used for COMP-2. Pictures are from one representative experiment. Magnification 40X.

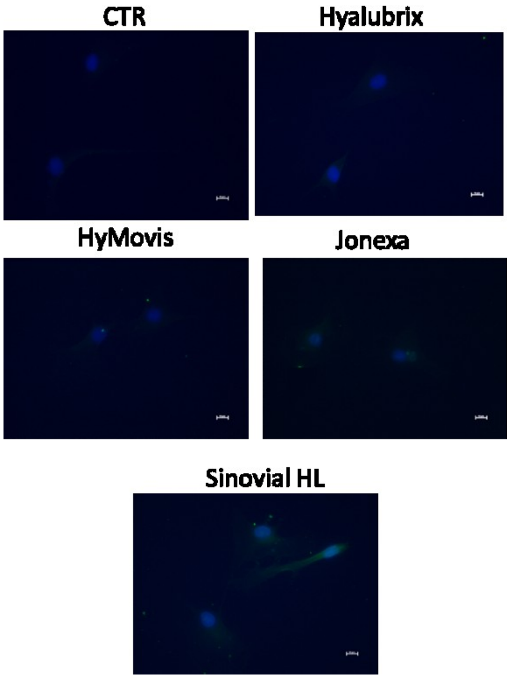

Figure S1 b. Immunofluorescence staining of HAS-1 in treated and untreated primary human synoviocytes. In the blue nuclei, a fitc-green antibody was used for HAS-1. Pictures are from one representative experiment. Magnification 40X.
